# A multidisciplinary systematic literature review on frailty: Overview of the methodology used by the Canadian Initiative on Frailty and Aging

**DOI:** 10.1186/1471-2288-9-68

**Published:** 2009-10-12

**Authors:** Sathya Karunananthan, Christina Wolfson, Howard Bergman, François Béland, David B Hogan

**Affiliations:** 1 Solidage Research Group, McGill University/Université de Montréal, Montreal, Canada; 2 Division of Clinical Epidemiology, McGill University Health Centre, Montreal, Canada; 3 Division of Geriatric Medicine, McGill University, Montreal, Canada; 4 Département d'administration de la santé, Université de Montréal, Montreal, Canada; 5 Division of Geriatric Medicine, University of Calgary, Calgary, Canada

## Abstract

**Background:**

Over the past two decades, there has been a substantial growth in the body of literature on frailty in older persons. However, there is no consensus on its definition or the criteria used to identify frailty. In response to this lack of consensus, the Canadian Initiative on Frailty and Aging carried out a set of systematic reviews of the literature in ten areas of frailty research: biological basis; social basis; prevalence; risk factors; impact; identification; prevention and management; environment and technology; health services; health and social policy. This paper describes the methodology that was developed for the systematic reviews.

**Methods:**

A Central Coordination Group (CCG) was responsible for developing the methodology. This involved the development of search strategies and keywords, article selection processes, quality assessment tools, and guidelines for the synthesis of results. Each review was conducted by two experts in the content area, with the assistance of methodologists and statisticians from the CCG.

**Results:**

Conducting a series of systematic literature reviews involving a range of disciplines on a concept that does not have a universally accepted definition posed several conceptual and methodological challenges. The most important conceptual challenge was determining what would qualify as literature on frailty. The methodological challenges arose from our goal of structuring a consistent methodology for reviewing literature from diverse fields of research. At the outset, certain methodological guidelines were deemed essential to ensure the validity of all the reviews. Nevertheless, it was equally important to permit flexibility in the application of the proposed methodology to capture the essence of frailty research within the given fields.

**Conclusion:**

The results of these reviews allowed us to establish the status of current knowledge on frailty and promote collaboration between disciplines. Conducting systematic literature reviews in health science that involve multiple disciplines is a mechanism to facilitate interdisciplinary collaboration and a more integrated understanding of health. This initiative highlighted the need for further methodological development in the performance of multidisciplinary systematic reviews.

## Background

While researchers, policy makers, administrators and health care providers generally agree that frailty can have an important impact on affected individuals, their families (particularly those involved in care-giving), the health care system and society as a whole [1], the concept of frailty remains controversial. Despite a substantial increase in the number of articles published on frailty over the past twenty-five years, there is no consensus on its definition or on what criteria should be used to identify older individuals with frailty [2]. Numerous models, definitions, criteria and approaches for its study have been advocated [3-7]. Nevertheless, few publications have systematically reviewed the quality of the evidence on frailty [8].

The Canadian Initiative on Frailty and Aging (CIFA) is an international partnership of researchers and health care providers formed in 2002, with the objectives of seeking to improve our understanding of the causes, trajectory and implications of frailty to thereby improve the lives of older persons at risk of frailty or with frailty by disseminating knowledge on its prevention, detection and treatment as well as the cost-effective organisation of services for those with frailty http://www.frail-fragile.ca/. To this end, the members of CIFA agreed that an essential early task for the group was to summarise the state of research on frailty in older persons in order to develop a working framework and identify research priorities for a program of enquiry.

As a first step, a qualitative review of existing models, definitions, and criteria for frailty was conducted and has been published elsewhere [2]. This work highlighted the need for an integrated interdisciplinary research approach and set the stage for a series of systematic literature reviews on particular aspects of frailty. The goal of these reviews was to collate, critically review and synthesise the current evidence on frailty across disciplines and to identify gaps in existing research.

Ten distinct reviews of pre-specified areas were conducted. The areas were selected by the CIFA Steering Committee with input from the broader group of CIFA investigators [see Additional file 1 for the list of CIFA investigators and members of the Steering Committee]. The areas and main research questions were: biological basis - what are the biological and physiological determinants of frailty?; social basis - how are social factors related to frailty over the life course?; prevalence - what is the prevalence of frailty in the community dwelling elderly?; risk factors - what factors have been shown to predict frailty?; impact of frailty - what impact does frailty have on affected individuals, their relatives, and the health care system?; clinical identification - what are the clinical operational diagnostic criteria to identify frailty?; prevention and management of frailty - can interventions aimed at the general population prevent frailty and/or its consequences?; use of environmental adaptations - what technological interventions have been demonstrated to increase quality of life and safety for frail older adults?; organisation of services - what are the integrated models of care delivery for the frail elderly?; and, health and social policy implications - what are the key policy issues in regard to care delivery and/or funding for the frail elderly?. Each area review was led by two to three CIFA investigators (referred to as Question Leaders) - see Table 1 for the list of Question Leaders and detailed research questions.

**Table 1:** Canadian Initiative on Frailty and Aging (CIFA) systematic literature review questions and investigators

Questions	Research Questions	Question Leaders
Biological basis	What are the biological and physiological determinants of frailty? How can these determinants be used to understand, define, predict and characterize frailty?	T. Fulop, MD PhD *Université de Sherbrooke* G. Duque, MD PhD *University of Sydney* D. Hogan, MD *University of Calgary*

Social basis	How has frailty been conceptualized from a social perspective? How are social factors related to frailty (as determinants, modifiers and consequences) over the life course?	M. Penning, PhD *University of Victoria* F. Béland, PhD *Université de Montréal*

Prevalence	What is the prevalence of frailty in the community dwelling elderly? Does prevalence vary by sex, age, ethnic group, availability of health services? How does prevalence vary according to the definitions used?	C. Wolfson, PhD *McGill University* H. Bergman, MD *McGill University*

Risk factors	What factors have been shown to predict frailty, functional decline, disability, mortality or increased resource utilization? What factors have been shown to predict successful aging?	G. Naglie, MD *University of Toronto* S. Gill, MD *Queen's University*

Impact on the individual, relatives and health services utilization	What impact does frailty have on affected individuals? What impact does frailty have on relatives of affected individuals? What impact does frailty have on the health care system?	B. Santos-Eggiman, MD PhD *Université de Lausanne* L. Seematter-Bagnoud, MD *Université de Lausanne*

Identification	What are the clinical operational diagnostic criteria? What are the tools for the screening and diagnosis, and investigation of frailty? Are there measures of severity of frailty?	S. Sternberg, MD *Maccabi Healthcare Services, Israel* A.M. Clarfield, MD *Ben Gurion University*

Prevention and Management	Can interventions aimed at the general population prevent frailty? Can interventions aimed at the general population prevent the consequences of frailty e.g. death, institutional admission, etc? Can interventions aimed at those who are frail or at risk of frailty, prevent the consequences of frailty?	C. Patterson, MD *McMaster University* J. Feightner, MD *University of Western Ontario*

Environment and Technology	What technological interventions have been demonstrated to increase quality of life and safety for frail older adults? Which technologies are not effective? What are the common characteristics of those technologies that have been found to be effective? What are the needs or opportunities for technologies to assist frail older adults and their caregivers that have not been adequately addressed?	G. Fernie, PhD *University of Toronto* B. Row, PhD *Toronto Rehabilitation Institute*

Health services	What are the integrated models of care delivery for the frail elderly? What are the trends in Canada on care delivery compared to the international literature? Are there comparative outcomes for different models of care, and to what extent have such models been evaluated? What are the common characteristics of the identified models of care and what are the consistencies of such characteristics?	M. Hollander, PhD *Hollander Analytical Services, Victoria* F. Béland, PhD *Université de Montréal*

Health and social policy	What are the key policy issues in regard to care delivery and/or funding for the frail elderly? What are the issues, alternatives and recommended solutions? What are the broader "meta" issues, which may be reflected in the set of policy issues? What recommendations can be made to decision-makers?	M. Hollander, PhD *Hollander Analytical Services, Victoria* N. Chappell, PhD *University of Victoria* M. Prince, PhD *University of Victoria*

The initial steps of the review process were completed between 2002 and 2005. The steps included all activities related to the literature searches, the article selection and the quality assessment. The results of the specific reviews are being published separately. Apart from the substantive results, an important part of the review process was the development of a methodology that would facilitate the conduct of systematic reviews of literature addressing multidisciplinary research questions and the summary of the literature on a concept lacking a widely accepted definition. This paper presents the methodology used for these systematic reviews and discusses some of the challenges faced as a result of the inconsistencies in the definition for frailty and the heterogeneity of research methods across the disciplines included.

## Methods

### Central Coordination Group

The Central Coordination Group (CCG) was based in Montreal, Quebec and was composed of CIFA investigators, with expertise in frailty and/or in research methodology, as well as staff hired for specific duties. The role of the CCG was to develop the methods that would be used for the systematic reviews and to assist Question Leaders in applying the methodology. CCG staff executed literature searches, retrieved and blinded abstracts and full articles. The quality assessment of articles was also conducted by the CCG if the Question Leaders did not have the local resources required to conduct these activities.

The systematic reviews were conducted by a multidisciplinary team of investigators with expertise in geriatrics, gerontology, epidemiology, sociology, biology, health services research, occupational therapy and rehabilitation engineering. During the review process, the CCG staff organised face-to-face and teleconference meetings, bringing together the Question Leaders for discussions on the review process and sharing of preliminary results. The primary objective of these meetings was to ensure consistency in the theoretical and methodological approach used for the reviews.

**Figure 1:**
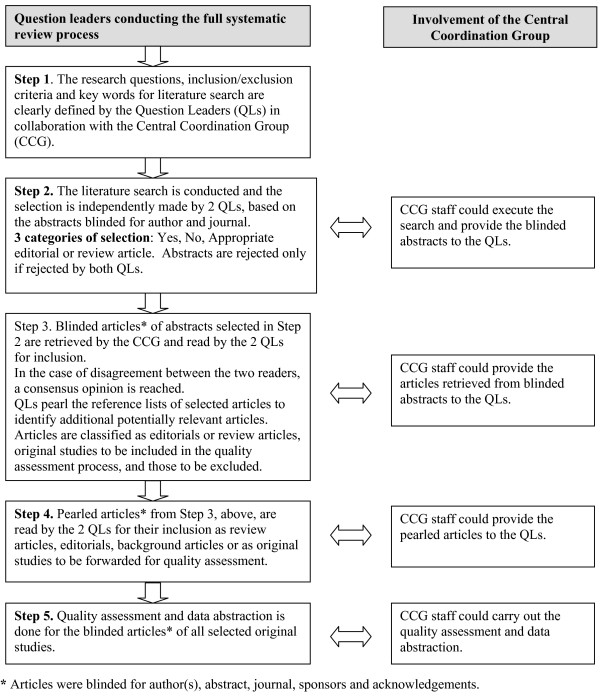
**Canadian Initiative on Frailty and Aging: general process for the systematic review of literature on frailty**.

The general process for the methodology is outlined in figure 1.

### Core Keywords for the Literature Search

As there is no widely accepted definition of frailty, the CIFA review strategies were inclusive and attempted to retrieve and examine all the literature relevant to the concept of frailty, rather than being limited to any single definition or model. Since most of the current definitions of frailty emphasize vulnerability to some adverse outcomes, CIFA reviews included literature on vulnerability [1]. Despite a growing consensus that frailty and disability are distinct concepts, several authors have used the terms synonymously [9]. In order not to miss this literature, the CIFA reviews also included papers on disability. Finally, since frailty has sometimes been described as the "flipside" of successful aging [2,10], this construct was also felt to be relevant to the reviews.

To ensure that all of these concepts were included within the CIFA reviews, the following general frailty-related terms were used as core keywords for *all *of the literature searches - 'aged' combined with any of the following terms: 'frail', 'frailty', 'vulnerable', 'vulnerability', 'successful aging', 'healthy aging', 'disability', or 'disabled persons'. For each Question, these core keywords were combined with review-specific keywords.

### Review Process

In the first step of the review, the Question Leaders refined the research questions to be addressed through their systematic review. They then identified review-specific keywords for the literature search, and decided upon the inclusion/exclusion criteria to be used in the selection of articles. The literature searches were conducted using MedLine and AgeLine, using the core frailty-related keywords combined with review-specific keywords. The searches were limited to publications in English or French, original research, and human studies. In accordance with the timing of the CIFA initiative, initial searches were conducted in the summer and fall of 2003 for papers published between 1997 and 2003. In the winter of 2005, the searches were updated to include literature published until the end of December 2004. All searches were overseen by a librarian with training and experience in the health sciences.

**Figure 2:**
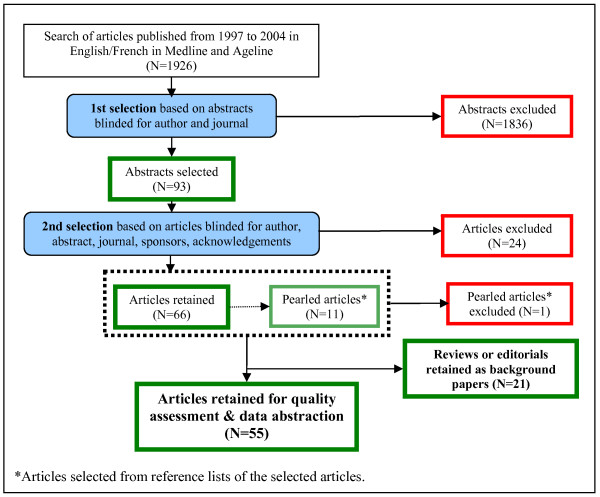
**Triage of articles for the systematic literature review on the prevalence of frailty**.

Following the literature search, the Question Leaders were sent the abstracts of the papers identified by the question specific search. The names of the authors and journal were removed from the abstracts. The two Question Leaders then independently reviewed each abstract for relevance to their assigned area. For abstracts that were selected as relevant by at least one of the Question Leaders, the full articles were retrieved. The Question Leaders then read through the full article, while remaining blinded for the author(s), abstract, journal, sponsors (if any) and acknowledgement (if any). They selected those studies that met the pre-specified inclusion criteria. Disagreements between Question Leaders were resolved through discussion until a consensus was reached. The references in each of the selected articles were then "pearled" (i.e., reviewed in an effort to find relevant papers not identified in the original searches) [11]. All identified relevant studies then underwent quality assessment and data abstraction. Pertinent editorials and review articles found during the literature search were retained as background papers. Figure 2 presents, as an illustration, the article selection flowchart for the review on the prevalence of frailty.

### Quality Assessment and Data Abstraction

All studies included in the review underwent quality assessment to evaluate how well the study design and study conduct reduced the potential for bias [12]. In addition to addressing issues specific to the study design (i.e. cohort; cross-sectional; randomised controlled trial), the quality assessments incorporated the unique characteristics specific to the review question. For the most part, we were unable to find quality assessment and data abstraction tools that fully met the requirements of our review [13]. For this reason, we adapted existing tools or designed new ones [14,15]. Based on discussions between the methodologists and Question Leaders, we developed the items that would be included in the tools for each of the Questions. The scale and format of the tools were then tested and modified based on feedback from the Quality Assessors. Depending upon the study design, our quality assessment examined between eleven and twenty-four items concerning specific methodological features of the study [see Additional file 2].

The items included in the methodological quality assessment tools varied according to the study design and what methodological features were being examined (e.g. internal validity, external validity, or reliability). For every tool, each item as well as the overall quality was rated on a scale of one to four (with four representing the highest quality). A global subjective ranking was also assigned by the Quality Assessors, to reflect the overall quality of the article considering the individual items that were rated. The assessors also indicated whether they felt the study was relevant to the systematic review. The data abstraction form used in the reviews was specific to the study design used, and included one section on the design features and another on results that were pertinent for the review.

The quality assessments and data abstractions were conducted by graduate students in epidemiology or a related discipline. Prior to being hired, the assessors were pre-tested to ensure that they had sufficient knowledge of epidemiological concepts. Once hired, they received a full day of training on the application of the CIFA quality assessment and data abstraction forms. A total of 16 assessors were involved in the quality assessments conducted by the CCG. Two assessors were randomly assigned to each article reviewed. They completed the assessment forms independently and then met to arrive at a consensus rating of individual items as well as on the overall methodological quality of the article. Data abstraction was completed by one of the two assessors.

### Classification

The authors of the included studies differed widely on what they meant by terms like frailty, disability, and vulnerability. For example, some defined frailty as being present when there were limitations in activities of daily living [16,17] or when participants were institutionalised [18,19]. Others required that distinct signs and symptoms be present in order to identify the presence of frailty [20].

After reviewing the first set of articles retrieved in our literature searches, we found that most author definitions for frailty fell into one of five broad categories: vulnerability - physiological syndrome; vulnerability - complex syndrome; vulnerability - geriatric syndrome; disability; and, setting/source of recruitment. To more clearly describe the studies included and permit subgroup analyses, we classified all of the studies reviewed based on the *definition *of frailty offered by the authors in the methods section of their article. Articles that did not fit into one of these categories were classified as "other" (see Table 2 for the definitions of the categories used).

**Table 2:** Classification categories for frailty literature

Category	Definition
**1. Vulnerability**	

***1.1 Physiological syndrome***	Operational criteria whereby vulnerability is based on physiological factors and/or physical functional impairments (performance measures); multiple systems have to be considered for a study to be classified as using a physiological syndrome definition for frailty.

***1.2 Complex syndrome***	Operational criteria whereby vulnerability is based on some combination of physiological and/or functional impairment (physical performance measures) with age, cognitive, psychological (e.g., depressive symptoms), sensory and/or social factors,

***1.3 Geriatric syndrome***	Operational criteria whereby vulnerability is based on some combination of chronic disease, the geriatric giants (such as falls, incontinence), functional impairment, disability, cognition, health service utilisation, and/or mortality risk

**2. Disability**	ADL/IADL measures with or without functional impairment

**3. Setting or source of recruitment**	Criteria refer to the setting or the source of recruitment e.g., all participants in a nursing home or all participants eligible for geriatric assessment

**4. Other**	The definition used does not fit into any of the above-mentioned categories

Two of the authors (H.B. and D.H.), both geriatrician-researchers, classified each article selected for the detailed review. For the first one hundred articles, the classifications were done independently followed by a consensus discussion in the event of discrepant classification. As the agreement was high, for the remaining articles only one of the two (H.B. or D.H.) carried out the classification. In the event of uncertainty, there was consultation that resulted in consensus.

Table 3 presents the final number of articles retained for review in each of the systematic reviews conducted, as well as the quality assessments and classification. The reviews on Health Services and Health and Social Policy followed a methodology that did not involve the same type of quality assessment and classification as the others, and so they have not been included in Table 3.

**Table 3:** Quality assessments and definitional classifications of articles selected for the reviews

Questions	Number of articles included for quality assessment	Quality Assessment		Classification of definition					
		
		1-2	3-4	Vulnerability Physio-logical	Vulnerability Complex	Vulnerability Geriatric	Disability	Setting/Source	Other
Biological basis	81	55	26	12	3	7	10	10	39

Social basis	74	9	65	1	7	4	62	0	0

Prevalence	55*	9	46	7	4	9	32	0	3

Risk factors	88	13	75	2	4	7	63	0	12

Impact	34	3	31	1	1	7	20	1	4

Identification	24	16	8	2	3	5	6	1	7

Prevention & Management	48*	4	44	1	0	11	4	14	18

Environment & Technology	65†	15	39	0	0	5	19	17	24

* These articles were rated on a scale from 1-3 (poor, fair, good). Articles that were rated "poor" are presented in the 1-2 category and articles rated as "fair" or "good" are presented in the 3-4 category.

† 11 of these articles had study designs that could not be evaluated with any of the CIFA quality assessment tools.

### Grades of Evidence

To summarise both the quality of the evidence and the direction of the findings, the Question Leaders used the categories presented in Table 4, assigning number and letter codes to the quality of the methodology and direction of results of each article respectively. For example, a study that was found to be of very good quality *and *presented clear evidence of a negative association was assigned a Grade of Evidence of 4B.

**Table 4:** Grades of evidence used to assess the frailty literature

Direction of association^§^	Methodological Quality^§^		
	
	1. Very Poor†	2. Poor†	3. Good
A. Clear evidence of positive association (i.e. increased risk)	**1A**	**2A**	3A

B. Clear evidence of negative association (i.e. protective, preventive)	**1B**	**2B**	3B

C. Absence of Evidence (i.e. H_0 _not rejected or wide CIs)	**1C**	**2C**	3C

D. Statistically significant association, but not clinically relevant	**1D**	**2D**	3D

§ Rows: Clarity and Direction of Evidence; Columns: Methodological Quality of the study

† The cells highlighted in bold do not provide good quality evidence on the question under study.

## Results

We conducted a series of systematic literature reviews that involved a range of disciplines on a concept that does not have a universally accepted definition. This endeavour posed several conceptual and methodological challenges.

By conducting a broad literature search, we retrieved a heterogeneous body of literature, and as a result it was not always appropriate or possible to compare results across studies. Grouping the studies according to the definition of frailty provided by the study author was a useful way of dealing with this heterogeneity. This approach allowed the reviewers to compare study results within a given definition of frailty. Nevertheless, a few Question Leaders chose to narrow down the final review to one or two definitions of frailty in order to make the process more manageable. For example for the review of risk factors for frailty, dealing with the numerous risk factors identified for the various different definitions of frailty created an unwieldy matrix of results that could not be summarized in a single review. For this reason the Question Leaders chose to limit themselves to one of the possible definitions for frailty. For other Questions, the use of a broad search contributed to noteworthy findings. For example, in the review of literature on the prevalence of frailty, there was marked variation in reported point-estimates, much of which was likely attributable to differences in the definitions, criteria and tools used to identify frailty. This underscored the observation that researchers are not speaking a common language when discussing frailty, and whichever definition one chooses to apply will have important effects on the findings [21].

The CIFA reviews examined research from different disciplines, requiring the review methodology to accommodate differences in disciplinary conceptions and research practices [22]. At the outset, certain methodological guidelines were deemed necessary to ensure the validity and reliability of all the reviews. Elements such as formulating a clear research question, predetermining the article inclusion criteria, having two independent reviewers selecting the articles, and blinding at all stages of article selection and quality assessment were crucial in ensuring the reproducibility of the review. In an attempt to be as comprehensive as possible in the reviews, literature searches were conducted in two different electronic databases and the reviewers pearled the references of selected articles to identify additional articles that may have been missed by the electronic searches. A librarian was involved in reviewing all the electronic search strategies and proposing complementary steps when the initial searches did not yield appropriate results. The CCG proposed limits in the timeframe and languages of the literature to be reviewed. These were selected to match the resources available. Similar limits were implemented across all the reviews in order to achieve some consistency.

On the other hand, tools for quality assessment and data abstraction as well as the presentation of results had to be developed to cater to the individual reviews and the type of study designs that were being reviewed. Nevertheless, a number of common guidelines were proposed. For example, it was decided that the quality assessment of the overall study would result in a subjective score. A contentious issue in quality assessment is the method of arriving at a score for the overall quality of the study. Some advocate a sum of all the individual components assessed. We felt that would not accurately reflect the overall quality of a study since all the items could not be weighted equally. In certain cases, a single flaw could be fatal to the validity of a study, and in other cases multiple minor flaws did not necessarily compromise the overall methodological quality of the study. Another guideline proposed by the CCG was that reviewers determine and present grades of evidence for their findings. The latter were useful for review questions that addressed association between risk factors and frailty or biological markers and frailty, but were not applicable to questions on the clinical identification of frailty or even the prevalence of frailty.

In some instances, the Question Leaders modified aspects of the proposed methodology to allow them to meet specific objectives. For example, some included articles that were published outside the proposed timeframe in order to incorporate seminal papers that were judged to still shape thinking about frailty in their field. While consistency in methodology was clearly desirable, it was important to permit flexibility in the application of the proposed methodology to capture the essence of frailty research within the given fields of research. All modifications to methodology were discussed and approved by the CIFA Steering Committee and CCG. Our main objective was to ensure that the methodology for each review could be clearly described (and replicated) and that the methodological choices were well aligned with the research questions.

Due to the complexity of the topic and process, as well as the number of investigators involved, the reviews took longer than expected. The development and fine-tuning of the methodology required lengthy discussions and consultations spanning several months. The quality assessment of an article for instance generally took between thirty minutes and two and a half hours per assessor, with an additional fifteen to thirty minutes for the consensus between assessors. Given the large number of articles assessed, this phase also involved a substantial amount of time. Further, since the quality assessors were not experts in the content areas, the Question Leaders sometimes deemed it necessary to modify the assessments, which led to some duplication of the work. Because the initial review process took so long, the reviews were behind schedule, and all the literature searches had to be updated by a year.

## Discussion

The most important conceptual challenge in conducting these systematic reviews was to determine what would qualify as being part of the literature on frailty. Some authors have treated frailty as synonymous with vulnerability, disability, or dependence, whereas others have attempted to describe frailty as a distinct concept [2,9]. The diversity in models is an indication of the isolation of researchers working on frailty [2]. Though this raised challenges, we elected to be inclusive in our literature review. We did not begin by debating the merits of the competing viewpoints and adopting a specific model and/or definition of frailty [23,24]. By being as inclusive as possible in our selection of literature, we felt that we could contribute to a more comprehensive understanding of the concept of frailty.

The methodological challenges faced in conducting these reviews arose from our goal of trying to establish and adhere to a consistent methodology for reviewing literature from diverse fields of research. While there is no standardised methodology for systematic literature reviews [25], the methodology described was developed by the CCG in order to maintain consistency and a high standard in the systematic reviews of frailty. Several groups have provided guidelines for conducting systematic reviews. Most of these are limited to systematic reviews and meta-analyses of randomised clinical trials (RCTs). However, the systematic review of research using other study designs is increasingly common and recognised as necessary for answering many research questions that are not amenable to RCTs. The CIFA reviews included cross-sectional, longitudinal and qualitative studies and required the development of a methodological approach that would be applicable to all of these.

One of our interests in conducting these reviews was to determine whether the approaches to frailty in the different fields are complementary and whether their findings converge. We elected to be inclusive in our literature review as the interest in frailty research spans a number of fields as diverse as geriatrics, gerontology, biology of aging, sociology, rehabilitation science, and health policy. Each has their own approach to the conceptualisation of frailty and addresses different research questions. Our intention was to foster a dialogue between researchers from different fields in order to stimulate more interdisciplinary collaborative work on frailty thereby establishing new avenues for research on the topic [26]. From this perspective the CIFA review process was extremely successful.

The literature searches for all reviews yielded extensive material on disability, but very limited results on frailty itself. Within the literature on frailty, there was substantial variation in the definition and operationalisation of frailty, even within a single area of research. Evidently, differences in the definitions had important effects when investigating such questions as the prevalence, risk factors, interventions, or impact of frailty. The results of the reviews suggest that there is a need and potential for evidence-based convergence in the definition of frailty across the various fields of research. The CIFA reviews have resulted in manuscripts in each of the ten areas. At the time of submission of the current paper, these manuscripts have either been submitted for publication or are in preparation for submission.

The results of the systematic reviews across the various disciplines point to exciting new horizons in the area of frailty research. Future work has the potential of furthering our understanding of the aging process and offers the hope that we can identify vulnerable older adults [1]. Despite the debate on the exact nature of frailty, there is no disagreement on its catastrophic impact on older individuals, their families (particularly those involved in providing support to the older individual), and on society as a whole. Ultimately, to be useful frailty research must lead to the identification of effective health promotion, prevention, treatment, rehabilitation, and care interventions. To this end, future research in frailty needs to consider existing knowledge across disciplines in order to work towards the development of a comprehensive framework of frailty that is relevant to clinicians, researchers and frail individuals.

By conducting a systematic literature review on frailty using a meticulous and explicit process that involved many different fields of research, CIFA has made an important contribution to frailty research. The results of the systematic reviews will enable us to establish the status of current knowledge, make well-founded suggestions for future research, and promote collaboration between disciplines.

Conducting systematic literature reviews in health science involving multiple disciplines is a first step towards more interdisciplinary collaboration as well as a more integrated understanding of health. However, such an endeavour is labour-intensive and requires a team with a high level of methodological and substantive expertise. This initiative also highlighted the need for developments in the area of multidisciplinary systematic reviews.

## Competing interests

The authors declare that they have no competing interests.

## Authors' contributions

SK was involved in the design and implementation of the systematic reviews as well as the drafting of the manuscript. CW, HB, FB, and DH were involved in the conception, design and implementation of the systematic reviews as well as the critical review of the manuscript. All authors read and approved the final manuscript.

## Pre-publication history

The pre-publication history for this paper can be accessed here:

http://www.biomedcentral.com/1471-2288/9/68/prepub

## Supplementary Material

Additional file 1 **Canadian Initiative on Frailty and Aging Investigators and Question Leaders**. Question Leaders and members of the Steering Committee for the Canadian Initiative on Frailty and Aging, along with their University affiliations. Click here for file

Additional file 2 **Quality assessment and data abstraction form for longitudinal studies on the biological basis of frailty**. The assessment tool used to the rate the quality of longitudinal studies for the systematic literature review on the biological basis of frailty. Click here for file

## References

[B1] Bergman H Ferrucci L Guralnik J Hogan DB Hummel S Karunananthan S Frailty: an emerging research and clinical paradigm issues and controversies J Gerontol A Biol Sci Med Sci 2007 62 731 737 17634320 10.1093/gerona/62.7.731 PMC2645660

[B2] Hogan DB MacKnight C Bergman H Models, definitions, and criteria of frailty Aging Clin Exp Res 2003 15 1 29 14580013

[B3] Fried LP Tangen CM Walston J Newman AB Hirsch C Gottdiener J Frailty in older adults: evidence for a phenotype J Gerontol A Biol Sci Med Sci 2001 56 M146 M156 11253156 10.1093/gerona/56.3.m146

[B4] Mitnitski AB Graham JJ Mogilner AE Rockwood K Frailty, fitness and late-life mortality in relation to chronological and biological age BMC Geriatr 2002 2 1 11 10.1186/1471-2318-2-1 11897015 PMC88955

[B5] Puts MT Lips P Deeg DJ Sex differences in the risk of frailty for mortality independent of disability and chronic diseases J Am Geriatr Soc 2005 53 40 47 10.1111/j.1532-5415.2005.53008.x 15667374

[B6] Strawbridge WJ Shema SJ Balfour JL Higby HR Kaplan GA Antecedents of frailty over three decades in an older cohort J Gerontol B Psychol Sci Soc Sci 1998 53 S9 16 9469175 10.1093/geronb/53b.1.s9

[B7] Schuurmans H Steverink N Lindenberg S Frieswijk N Slaets JP Old or frail: what tells us more? J Gerontol A Biol Sci Med Sci 2004 59 M962 M965 15472162 10.1093/gerona/59.9.m962

[B8] Wells JL Seabrook JA Stolee P Borrie MJ Knoefel F State of the art in geriatric rehabilitation. Part I: review of frailty and comprehensive geriatric assessment Arch Phys Med Rehabil 2003 84 890 897 10.1016/S0003-9993(02)04929-8 12808544

[B9] Fried LP Ferrucci L Darer J Williamson JD Anderson G Untangling the Concepts of Disability, Frailty, and Comorbidity: Implications for Improved Targeting and Care J Gerontol A Biol Sci Med Sci 2004 59 M255 M263 10.1093/gerona/59.3.m255 15031310

[B10] Hamerman D Toward an understanding of frailty Ann Intern Med 1999 130 945 950 10375351 10.7326/0003-4819-130-11-199906010-00022

[B11] Greenhalgh T Peacock R Effectiveness and efficiency of search methods in systematic reviews of complex evidence: audit of primary sources BMJ 2005 331 1064 1065 10.1136/bmj.38636.593461.68 16230312 PMC1283190

[B12] West S King V Carey TS Lohr KN McKoy N Sutton SF Systems to Rate the Strength of Scientific Evidence Evidence Report/Technology Assessment 2002 47 PMC4781591 11979732

[B13] Norris SL Atkins D Challenges in using nonrandomized studies in systematic reviews of treatment interventions Ann Intern Med 2005 142 1112 1119 15968036 10.7326/0003-4819-142-12_part_2-200506211-00011

[B14] Downs SH Black N The feasibility of creating a checklist for the assessment of the methodological quality both of randomised and non-randomised studies of health care interventions J Epidemiol Community Health 1998 52 377 384 10.1136/jech.52.6.377 9764259 PMC1756728

[B15] Jadad AR Moore RA Carroll D Jenkinson C Reynolds DJ Gavaghan DJ Assessing the quality of reports of randomized clinical trials: is blinding necessary? Control Clin Trials 1996 17 1 12 10.1016/0197-2456(95)00134-4 8721797

[B16] Tomita MR Mann WC Fraas LF Burns LL Racial differences of frail elders in assistive technology Assistive Technology 1997 9 140 151 10177451 10.1080/10400435.1997.10132305

[B17] Malcolm M Mann WC Tomita MR Fraas LF Stanton KM Gutlin L Computer and Internet use in physically frail elders Phys Occup Ther Geriatr 2001 19 15 32 10.1300/J148v19n03_02

[B18] Breuer B Trungold S Martucci C Wallenstein S Likourezos A Libow LS Relationships of sex hormone levels to dependence in activities of daily living in the frail elderly Maturitas 2001 39 147 159 10.1016/S0378-5122(01)00208-0 11514113

[B19] Morrison MF Redei E TenHave T Parmelee P Boyce AA Sinha PS Dehydroepiandrosterone sulfate and psychiatric measures in a frail, elderly residential care population Biol Psychiatry 2000 47 144 150 10.1016/S0006-3223(99)00099-2 10664831

[B20] Fried LP Walston J Hazzard WR, Blass JP, Halter JB, Ouslander JG, Tinetti ME Frailty and failure to thrive Principles of geriatric medicine and gerontology 2003 New York: McGraw Hill 1487 1502

[B21] van Iersel MB Rikkert MG Frailty criteria give heterogeneous results when applied in clinical practice J Am Geriatr Soc 2006 54 728 729 10.1111/j.1532-5415.2006.00668_14.x 16686901

[B22] Couturier Y Dumas-Laverdiere C Comparison of Methods & Interdisciplinary Possibilities. The Case of Literature Reviews in Social Work and in Nursing Sciences The Qualitative Review 2006 11 80 87

[B23] Bergman H The Canadian Initiative on Frailty and Aging Aging Clin Exp Res 2003 15 1 214580013

[B24] Rockwood K What would make a definition of frailty successful? Age Ageing 2005 34 432 434 10.1093/ageing/afi146 16107450

[B25] Klassen TP Jadad AR Moher D Guides for reading and interpreting systematic reviews: I. Getting started Arch Pediatr Adolesc Med 1998 152 700 704 9667544

[B26] Tinetti ME Fried T The end of the disease era Am J Med 2004 116 179 185 10.1016/j.amjmed.2003.09.031 14749162

