# Supplementary material for: A multidisciplinary systematic literature review on frailty: Overview of the methodology used by the Canadian Initiative on Frailty and Aging
Source: BMC Med Res Methodol. 2009 Oct 12;9:68. doi: 10.1186/1471-2288-9-68 (PMC2765448; doi:10.1186/1471-2288-9-68)
Supplement: Additional file 1 — Canadian Initiative on Frailty and Aging Investigators and Question Leaders. Question Leaders and members of the Steering Committee for the Canadian Initiative on Frailty and Aging, along with their University affiliations. [file 1471-2288-9-68-S1.DOC]

Additional file 1:

Canadian Initiative on Frailty and Aging Investigators and Question Leaders:

| **Question Leaders** |
| --- |
| F. Béland, PhD *Université de Montréal, Montreal, Canada*  H. Bergman, MD *McGill University, Montreal, Canada*  N. Chappell, PhD *University of Victoria, Victoria, Canada*  A.M. Clarfield, MD *Ben Gurion University, Beersheva, Israel*  G. Duque, MD PhD *University of Sydney, Sydney, Australia*  J. Feightner, MD *University of Western Ontario, London, Canada*  G. Fernie, PhD *University of Toronto, Toronto, Canada*  T. Fulop, MD PhD *Université de Sherbrooke, Sherbrooke, Canada*  S. Gill, MD *Queen’s University, Kingston, Canada*  D. Hogan, MD *University of Calgary, Calgary, Canada*  M. Hollander, PhD *Hollander Analytical Services, Victoria, Canada*  G. Naglie, MD *University of Toronto, Toronto, Canada*  C. Patterson, MD *McMaster University, Hamilton, Canada*  M. Penning, PhD *University of Victoria, Victoria, Canada*  M. Prince,PhD *University of Victoria, Victoria, Canada*  B. Row, PhD *Toronto Rehabilitation Institute, Toronto, Canada*  B. Santos-Eggiman, MD PhD *Université de Lausanne, Lausanne, Switzerland*  L. Seematter-Bagnoud, MD *Université de Lausanne, Lausanne, Switzerland*  S. Sternberg, MD *Maccabi Healthcare Services, Jerusalem, Israel*  C. Wolfson, PhD *McGill University, Montreal, Canada* |
| **Steering Committee**: |
| H. Bergman, MD *McGill University, Montreal, Canada* C. Wolfson, PhD *McGill University, Montreal, Canada* D. Hogan, MD *University of Calgary, Calgary, Canada* F. Béland, PhD *Universite de Montréal, Montreal, Canada* J. W. Feightner, MD *University of Western Ontario, London, Canada* G. Fernie, PhD *University of Toronto, Toronto, Canada* C. MacKnight, MD *Dalhousie University, Halifax, Canada* F. Paccaud, PhD *University of Lausanne, Lausanne, Switzerland*  C. Patterson, MD *McMaster University, Hamilton, Canada* |
