# Supplementary material for: A multidisciplinary systematic literature review on frailty: Overview of the methodology used by the Canadian Initiative on Frailty and Aging
Source: BMC Med Res Methodol. 2009 Oct 12;9:68. doi: 10.1186/1471-2288-9-68 (PMC2765448; doi:10.1186/1471-2288-9-68)
Supplement: Additional file 2 — Quality assessment and data abstraction form for longitudinal studies on the biological basis of frailty. The assessment tool used to the rate the quality of longitudinal studies for the systematic literature review on the biological basis of frailty. [file 1471-2288-9-68-S2.DOC]

Additional file 2:

Quality assessment and data abstraction form for longitudinal studies on the biological basis of frailty.

| QUALITY CRITERIA* | DATA ABStraction: METHODS |
| --- | --- |
| 1. Do the authors clearly describe the population from which the participants were drawn? | Study Population:  Database:  Which waves/years of data collection? |
| 1. Are the inclusion/exclusion criteria 2. clearly described? 3. appropriate for the question?   e.g. were important groups excluded? | Inclusion/Exclusion criteria: |
| 1. Are the participants in the study representative of the population from which they were recruited? | Description of study participants:  Age at baseline (mean, range, median):  Age at follow-up (mean, range, median): |
| 1. Did the authors ensure comparability of groups by addressing issues of: 2. Selection from same source population? 3. Participation rates by exposure? 4. Differential losses to follow-up? 5. Adherence/Crossovers/ Contamination? | Characteristics of different comparison groups:  Intervention (exposed) group:  Number lost to follow-up because of death?  Number lost to follow-up for reasons other than death?  Comparison group:  Number lost to follow-up because of death?  Number lost to follow-up for reasons other than death? |
| 1. Were the study participants in different comparison groups recruited over the same time period? | Recruitment time period: |
| 1. Was the outcome defined clearly? | Definition of the outcome(s): How was outcome measured?🞎 self-report 🞎 proxy observation🞎 clinical test 🞎 medical recordWas outcome measured the same way at all follow-up points?Was the outcome measured at baseline?🞎 Yes 🞎 No |
| 1. Were the outcome measures selected    1. Reliable?    2. Valid?    3. Reproducible? | Psychometric properties of the measures of outcome(s): |
| 1. Were the measures of exposures    1. Reliable?    2. Valid? | Measures of exposures: |
| 1. Were those measuring the main outcome blind to exposure status? | Blinding: |
| 1. Were measures exposures ascertained in the appropriate time frame (and repeated when necessary)? | Assessment timeframe of exposures: |
| 1. Were potential confounders measured and adequately addressed in the analysis? | Confounders measured: |
| 1. Was the follow-up long enough to detect causal relationships? | Length of follow-up (mean, median and range of follow-up): |
| 1. Were missing data handled appropriately? (i.e. case deletion, imputation methods)- | Missing data: |
| 1. Was a reasonable proportion of the baseline sample maintained until follow-up? | Overall participation rate at baseline:  Proportion of sample lost to follow-up: |
| 1. a) Was the statistical analysis appropriate?   b) Did the analytic techniques take into account different lengths of follow-up of participants? | Statistical analysis:  Multivariable analysis: 🞎Yes 🞎No  Adjustment for confounders (Which?):  If outcome measure was assessed at baseline, was there an adjustment for the baseline assessment?  By risk adjustment, stratification, exclusion? |
| 1. Did the study have sufficient power to detect statistically and/or clinically meaningful differences in the outcomes? | Sample size for different analyses and subgroups:  Proportion of the sample ≥ 65 years: |

* Each item in the question was rated on a scale of one to four, with four being the highest quality assessment
